# Bridging Rare to Common Diseases: Precision Medicine and the Transforming Landscape of Pediatric Allergy and Immunology

**DOI:** 10.1111/pai.70407

**Published:** 2026-06-21

**Authors:** Riccardo Castagnoli, Martina Votto, Ivan Taietti, Maria Isabel Delgado‐Dolset, Rubén Fernández‐Santamaría, Martha Jimenez Freites, Mattia Giovannini, Mark Kačar, Aspasia Karavelia, Janice Layhadi, Urszula Radzikowska, Cezmi A. Akdis, Mubeccel Akdis, Marketa Bloomfield, Francesco Cinetto, Antoine Deschildre, Jonathan Hourihane, Susanne Lau, Antonio Nieto‐Garcia, Ismail Ogulur, Liam O'Mahony, Mohamed Shamji, Milena Sokolowska, Maria Jose Torres, Sophia Tsabouri, Philippe Eigenmann, the EAACI‐Clemens von Pirquet Foundation

**Affiliations:** ^1^ Pediatric Unit, Department of Clinical, Surgical, Diagnostic, and Pediatric Sciences University of Pavia Pavia Italy; ^2^ Pediatric Clinic Fondazione IRCCS Policlinico San Matteo Pavia Italy; ^3^ Maternity and Pediatric Department Azienda Sanitaria Locale Benevento Italy; ^4^ Swiss Institute of Allergy and Asthma Research (SIAF) University of Zurich Davos Switzerland; ^5^ Immunology Department, IIS‐Fundación Jiménez Díaz Hospital Universitario Fundación Jiménez Díaz Madrid Spain; ^6^ Pediatric Department, Pediatric Allergy Out‐Clinics Consorci Sanitari Alt Penedès‐Garraf Barcelona Spain; ^7^ Pediatric Allergology and Clinical Immunology Department Hospital Sant Joan de Déu Barcelona Spain; ^8^ Allergy Unit Meyer Children's Hospital IRCCS Florence Italy; ^9^ Department of Health Sciences University of Florence Florence Italy; ^10^ Division of Allergy University Clinic of Respiratory and Allergic Diseases Golnik Slovenia; ^11^ ENT Department General Hospital of Kalamata Antikalamos Greece; ^12^ National Heart and Lung Institute Imperial College London London UK; ^13^ Department of Immunology, 2nd Faculty of Medicine Motol and Homolka University Hospital, Charles University Prague Czech Republic; ^14^ Department of Pediatrics, 1st Faculty of Medicine Thomayer's University Hospital, Charles University Prague Czech Republic; ^15^ Department of Medicine—DIMED Rare Diseases Referral Center, Ca' Foncello Hospital, University of Padova, AULSS2 Marca Trevigiana Treviso Italy; ^16^ CHU Lille, Service de Pneumologie et Allergologie Pédiatriques Hôpital Jeanne de Flandre, Univ Lille Lille France; ^17^ Paediatrics and Child Health Royal College of Surgeons in Ireland Dublin Ireland; ^18^ Children's Health Ireland Dublin Ireland; ^19^ Department of Pediatric Respiratory Medicine, Immunology and Critical Care Medicine Charité Universitätsmedizin Berlin Berlin Germany; ^20^ Pediatric Pulmonology and Allergy Unit Health Research Institute La Fe, Hospital Universitari i Politècnic La Fe Valencia Spain; ^21^ APC Microbiome Ireland University College Cork Cork Ireland; ^22^ School of Microbiology University College Cork Cork Ireland; ^23^ Department of Medicine University College Cork Cork Ireland; ^24^ Allergy Unit, Department of Medicine and Dermatology UMA, Hospital Regional Universitario de Málaga‐IBIMA Málaga Spain; ^25^ Child Health Department, Medical School University of Ioannina Ioannina Greece; ^26^ Pediatric Allergy Unit, Department of Pediatrics, Gynecology and Obstetrics University Hospitals of Geneva Geneva Switzerland

**Keywords:** allergy, biologics, inborn errors of immunity (IEI), pediatrics, precision medicine, primary atopic disorders (PAD), small molecules

## Abstract

Pediatric immunological and allergic conditions represent a broad spectrum, ranging from highly prevalent polygenic disorders (e.g., food allergy, atopic dermatitis, asthma, and allergic rhinitis) to rarer monogenic primary atopic disorders. The management of these conditions has undergone a paradigm shift in recent years. Moving away from a “one‐size‐fits‐all” approach, precision medicine aims to deliver the right treatment to the right patient at the right time. By integrating clinical phenotypes with molecular endotypes and using specific biomarkers and “Omics” techniques, scientists and clinicians can now employ targeted biological therapies that significantly improve patient outcomes. By identifying early therapeutic windows and specific biomarkers, pediatric specialists can implement personalized interventions that halt the atopic march and mitigate the global burden of chronic allergic diseases. By shifting the focus from symptom management to the neutralization of specific molecular pathways, it is now possible to achieve better disease control, reduce side effects associated with broad‐spectrum treatments such as systemic corticosteroids, and improve the quality of life for patients with refractory allergic diseases. This review, generated during a seminar funded by the Clemens von Pirquet Foundation, aims to delineate the “rare to common” pipeline, illustrating how precision medicine tools, including multi‐omics, biomarkers, and artificial intelligence methods, can connect mechanistic pathways across the immunological spectrum.

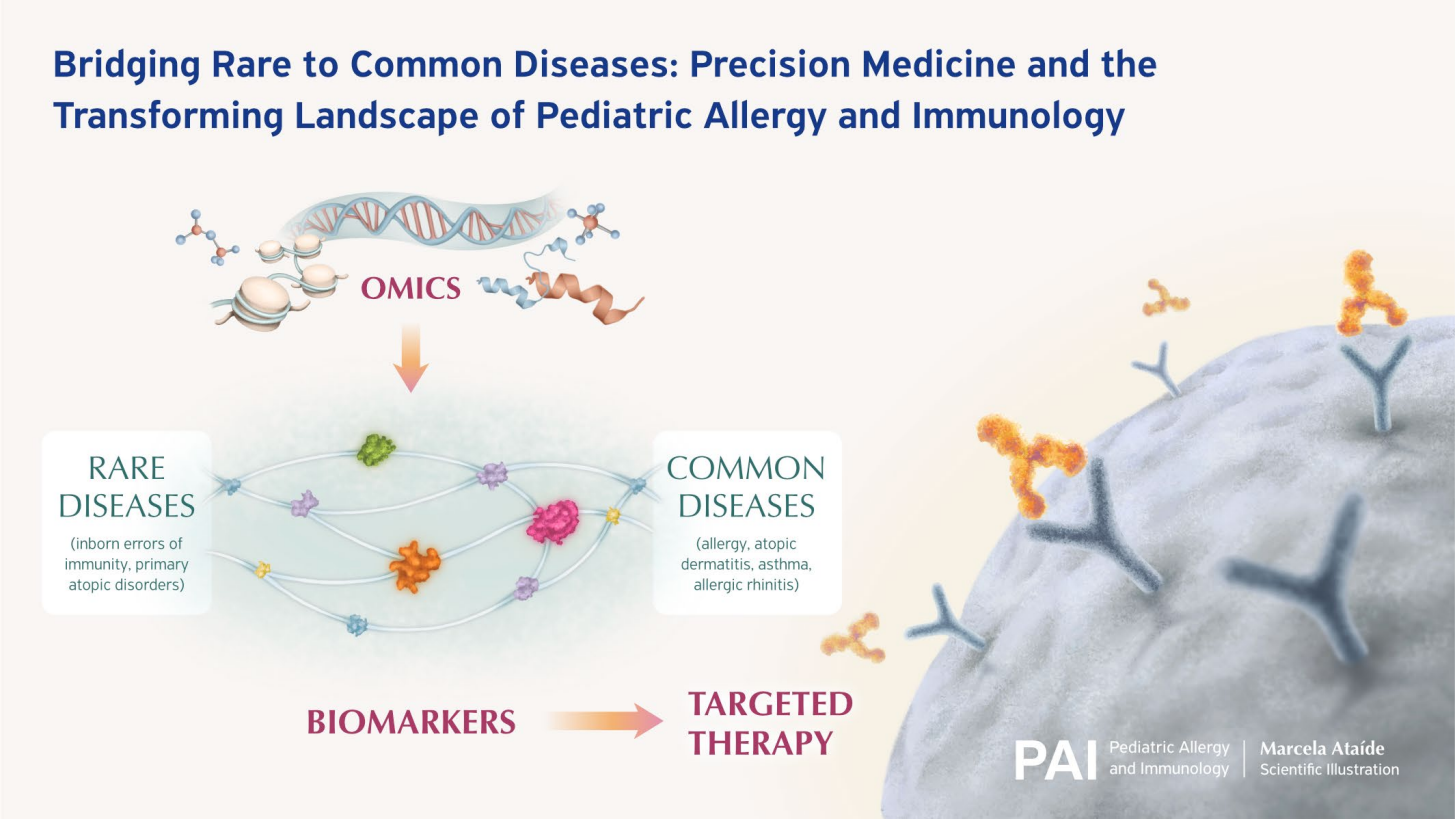

## Graphical abstract

This graphical abstract illustrates the conceptual framework linking rare and common diseases in pediatric allergy and immunology through precision medicine. On the left, rare conditions such as inborn errors of immunity and primary atopic disorders represent well‐defined monogenic models that reveal fundamental mechanisms of immune dysregulation. On the right, common allergic diseases—including asthma, atopic dermatitis, food allergy, and allergic rhinitis—are depicted as complex, polygenic disorders sharing overlapping pathogenic pathways. At the center, multi‐omics technologies (genomics, transcriptomics, proteomics, and metabolomics) enable the identification of shared molecular mechanisms and disease endotypes across this spectrum. These approaches facilitate the discovery of clinically relevant biomarkers that allow patient stratification based on underlying biology rather than clinical phenotype alone. The transition from biomarkers to targeted therapies highlights the application of precision medicine, where biologics and small molecules are selected to specifically modulate key immune pathways, such as type 2 inflammation. Overall, the figure emphasizes the “rare‐to‐common” translational pipeline, demonstrating how insights derived from rare monogenic diseases inform the understanding, diagnosis, and treatment of more prevalent allergic conditions, while also supporting the bidirectional repurposing of targeted therapies across the disease spectrum, ultimately improving patient outcomes through personalized therapeutic strategies.

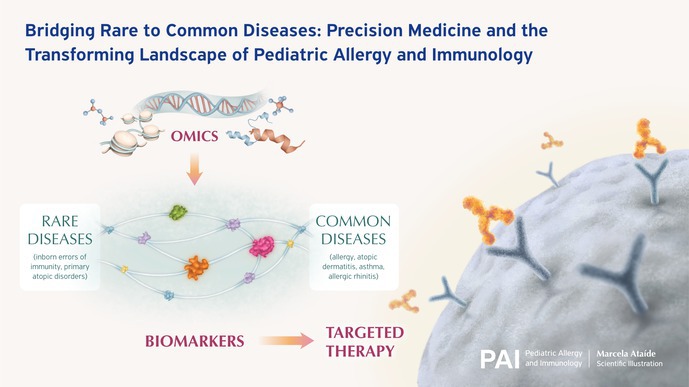

**Key message**

This work illustrates how bridging rare and common diseases advances precision medicine in pediatric allergy and immunology. Integrating shared mechanisms, biomarkers, and targeted therapies enables patient stratification and supports the repurposing of drugs across the disease spectrum to improve outcomes.

## 1 INTRODUCTION

Immuno‐allergic diseases exhibit high heterogeneity, ranging from highly common (e.g., asthma, atopic dermatitis [AD], food allergy [FA], and allergic rhinitis [AR]) to rare conditions (e.g., primary atopic disorders [PAD]). Immuno‐allergic conditions are no longer viewed as static entities, but rather as dynamic processes that evolve from infancy through adulthood. While the atopic march is the traditional model for this progression, precision medicine utilizes complex mathematical modeling to redefine these trajectories. Precision medicine transitions from a cross‐sectional snapshot (treating patients based on their current state) to a longitudinal continuum that starts early in life and evolves into irreversible structural changes. By identifying critical therapeutic timeframes and specific disease endotypes, pediatric allergists and immunologists can personalize the use of novel targeted biological therapies to potentially halt the inflammatory cascade before systemic comorbidities become entrenched.

Modern allergology and clinical immunology are increasingly guided by biomarkers, measurable indicators of biological states that can predict disease risk and trajectory. From rare *inborn errors of immunity* (*IEI*) to common allergies, biomarkers help link crucial mechanistic pathways across this spectrum, paving the way for *precision medicine* approaches.

A previous publication, supported by the Clemens von Pirquet Foundation, focused on understanding the trajectories of allergic disease and their importance in shaping the natural history and chronic consequences.^1^ In this review, we sought to link rare and common immunological and atopic diseases, starting with the understanding of the underlying pathogenetic mechanisms, highlighting similarities and differences, and ending with the use of translational research tools to personalize therapies.

## 2 INBORN ERRORS OF IMMUNITY: THE PARADIGM OF BRIDGING RARE TO COMMON DISEASES

IEI, formerly known as primary immunodeficiencies, represent an ever‐expanding group of more than 500 monogenic disorders with a remarkable diversity of clinical manifestations.^2^ Traditionally considered in the context of recurrent, severe, or opportunistic infections, IEI are now recognized as complex disorders that may also present with autoimmunity, autoinflammation, lymphoproliferation, malignancy, and, increasingly, severe allergic or atopic features.^3^ This broadened spectrum reflects significant advances in immunogenetics, high‐throughput sequencing, and systems biology, which have enabled the identification of novel molecular mechanisms and new disease entities.

Among the most intriguing developments of the last decade is the definition of PAD, a subset of IEI characterized by genetic defects that lead to dysregulated type 2 (T2) immune responses, excessive IgE production, eosinophilia, and/or aberrant mast cell activation.^4^ Clinically, PAD may manifest with severe AD, asthma, FA, and eosinophilic gastrointestinal disorders, often associated with immune dysregulation such as autoimmunity, lymphoproliferation, or increased susceptibility to infections.^4^ Importantly, these features can mimic common allergic diseases, leading to underrecognition or delayed diagnosis, yet PAD typically presents with very early onset, treatment resistance, and multiorgan involvement.^4^, ^5^ Classic examples of IEI with atopic phenotypes include hyper‐IgE syndromes (HIES), Wiskott–Aldrich syndrome (WAS), Omenn syndrome, immune dysregulation‐polyendocrinopathy‐enteropathy‐X‐linked (IPEX) syndrome, and IPEX‐like disorders, while recently reported entities such as the CBM‐opathies, affecting the caspase recruitment domain family member 11 (CARD11)‐B cell CLL/lymphoma 10 (BCL10)‐MALT1 paracaspase (MALT1) [CBM] signalosome complex, and the *STAT6* gain‐of‐function autosomal dominant disease continue to expand this spectrum.^6^, ^7^ These conditions challenge the traditional distinction between allergy and immunodeficiency, demonstrating how both may arise from a common underlying defect in immune regulation.^8^ Moreover, common variable immunodeficiency (CVID) serves as a bridge, representing a spectrum of diseases in which some have a clear monogenic cause, while others involve complex polygenic and epigenetic influences. Although the 2024 International Union of Immunological Societies classification has not yet formally grouped PAD into a distinct category, expert opinion increasingly supports recognizing IEI with predominant atopic manifestations as a specific subgroup within immune dysregulation disorders.^1^, ^8^, ^9^

The diagnosis of PAD requires heightened clinical awareness and the recognition of red flags such as very early onset of severe atopic disease, unusual infections, growth failure, multiorgan involvement, and/or a suggestive family history. Laboratory work‐up should include immunophenotyping, immunoglobulin levels, assessment of vaccine antibody responses, and markers of immune dysregulation, whereas genetic testing remains essential for definitive diagnosis. Functional assays may further clarify the pathogenic role of variants with uncertain significance and guide therapeutic decisions.^8^, ^9^

From a therapeutic standpoint, knowledge of the disrupted pathway has direct clinical implications. Hematopoietic stem cell transplantation or gene therapy may be curative in selected cases, whereas others can benefit from targeted biological therapies. T2‐directed biologics, such as anti‐interleukin (IL)‐4 receptor alpha or anti‐IL‐5/IL‐5 receptor monoclonal antibodies, and Janus kinase inhibitors (JAKi), such as ruxolitinib, baricitinib, and tofacitinib, are increasingly being evaluated in patients with PAD who have severe atopic manifestations refractory to conventional treatments. These advances exemplify how precision medicine can transform the management of patients with rare immunogenetic conditions.^10^

Ultimately, the study of rare monogenic allergic phenotypes offers dual benefits: it enables clinicians to provide life‐saving diagnoses and treatments for affected individuals while also providing fundamental insights into the pathogenesis of common allergic diseases. The evolving fields of IEI and PAD illustrate how integrating clinical expertise, translational research, and genomic discovery can reshape our understanding of immune‐mediated diseases and pave the way for a truly personalized medical approach in allergy and clinical immunology.

## 3 MECHANISTIC INSIGHTS INTO HIGHLY PREVALENT IMMUNE‐MEDIATED DISEASES

Highly prevalent immune‐mediated diseases such as asthma, allergic rhinitis, AD, and FA are characterized by dysregulated immune responses that involve complex interactions between genetic predispositions and environmental exposures.^11^ These conditions, often grouped under the umbrella term of allergic or T2 immune diseases, are driven primarily by exaggerated responses to harmless environmental antigens, such as pollen, dust mites, or food proteins. Central to their pathogenesis is the inappropriate activation of type 2 innate lymphoid cells (ILC2s) and helper T cells (Th2).^12^ These cells secrete key cytokines, such as IL‐4, IL‐5, and IL‐13, that orchestrate a cascade of events, including IgE class‐switching, eosinophil recruitment, and disruption of epithelial barriers, all of which contribute to inflammation and clinical symptoms. In asthma, it leads to airway hyper‐responsiveness, bronchial remodeling, and chronic inflammation, while in AD and eosinophilic esophagitis (EoE), impaired epithelial barrier function allows allergens and microbes to penetrate, triggering immune activation.^13^ Similarly, in FAs, the gut's immune system misidentifies dietary proteins as threats, leading to IgE‐mediated hypersensitivity and, in severe cases, anaphylaxis.

Affecting over 300 million individuals worldwide, asthma is a chronic respiratory condition of the airway characterized by episodes of wheezing, breathlessness, coughing, and chest tightness.^14^ It can be broadly classified into T2‐high and T2‐low endotypes, with T2‐high asthma accounting for 80% to 85% of school‐aged children and adolescents with asthma, including those with severe asthma.^15^ The two endotypes differ significantly in their underlying immunological pathways and epithelial barrier characteristics.^16^ T2‐low asthma is characterized by neutrophilic inflammation and Th1/Th17 response, whereas T2‐high asthma is mainly driven by eosinophils, mast cells, ILC2s, and Th2 cells.^16^, ^17^ The T2‐high asthma begins in the airway epithelium upon exposure to environmental factors such as allergens, pollutants, or viruses, leading to the production of alarmins, including thymic stromal lymphopoietin (TSLP), IL‐25, and IL‐33, chemokines, lipid mediators, and other active pro‐inflammatory cytokines, such as IL‐1β and GM‐CSF.^18^ The production of alarmins activates dendritic cells (DCs), which subsequently activate ILC2s and drive Th2 cell differentiation, both of which are responsible for the large production of Th2 cytokines.^19^, ^20^ Th2 cytokines are essential in driving the pathophysiology of asthma, with IL‐4 driving class switching of B cells to IgE‐producing plasma cells, IL‐5 driving the development, recruitment, and activation of eosinophils, and IL‐13 driving disease progression by accelerating tissue remodeling.^19^, ^20^, ^21^, ^22^ Furthermore, IL‐13 stimulation of the airway epithelium results in the generation of hypersecretory MUC5 AC‐expressing mucus cells, thereby impairing mucociliary clearance and predisposing to microbial superinfection and decreased host defense.^23^ Finally, studies have shown an alteration in the innate antimicrobial immune responses of the bronchial epithelium. Susceptibility to viruses, particularly rhinoviruses, which are the main triggers of asthma exacerbations, characterizes allergic asthma in children.^24^

Like asthma, FA is a serious public health concern that affects over 200 million people globally.^25^ FA often emerges in early childhood, with reactions to cow's milk, eggs, and peanuts being the most common FAs.^26^ Due to the rapid onset of symptoms, it has been speculated that both genetic and environmental factors are essential.^27^ When early oral exposure to foods is associated with tolerance, skin barrier impairment related to exposure to pollutants, detergents, and infections, as well as to genetic factors (e.g., filaggrin, SPINK5, and loricrin gene mutations), with or without AD, is a key player in FA development.^28^ Mechanistically, FA shares similarities with asthma, involving antigen presentation by DCs and the activation of Th2 and T follicular helper (Tfh) cells. Upon re‐exposure, allergens are recognized by sIgE, which bind high‐affinity receptors on basophils and mast cells, triggering degranulation and the release of histamine, cytokines, leukotrienes, and prostaglandins. This can result in immediate hypersensitivity reactions or even life‐threatening anaphylaxis.^27^

AD is the most common inflammatory condition of the skin, and through reduced skin barrier function, AD often precedes the onset of asthma, FA, and AR. A key driver of the pathobiology of AD is epithelial barrier dysfunction, including filaggrin (*FLG*) deficiency, a lack of certain epidermal lipids and fatty acids, and tight‐junction abnormalities.^23^ Loss‐of‐function (LOF) mutations in the *FLG* gene are strongly associated with enhanced allergen presentation, early onset, and a more severe phenotype, and define a phenotype prone to atopic comorbidities.^24^, ^25^ However, since *FLG* mutations are only found in a minority of AD patients, dysregulated immune responses are likely critical in driving skin barrier dysfunction in most AD sufferers.^26^, ^27^ AD is associated with a dominant T2‐mediated immune response that involves ILC2s, Th2, and mast cells, associated with alarmins and T2 cytokines. DC‐derived IL‐23 drives the differentiation of Th17 and Th22 cells, which subsequently produce IL‐22, which inhibits keratinocyte differentiation, thereby amplifying disease progression in AD.

Genetics plays a significant role in predisposing individuals to these conditions. Variants in FLG impair skin barrier integrity, increasing susceptibility to environmental allergens and subsequent sensitization.^29^ The clinical relevance of *FLG* variants has also been demonstrated in FA, where environmental peanut exposure strongly correlates with sensitization in children with AD and *FLG* LOF mutations.^30^ However, *FLG* variants in adults without concomitant AD are not associated with increased allergen sensitization.^31^ Other genes include polymorphisms in *TSLP*, *SPINK5*, and corneodesmosin, which are linked to AD and FA.^32^, ^33^ Genes involved in immune regulation, such as *IL4, IL13*, and *STAT6*, have been associated with heightened Th2 responses and increased risk of asthma and FA. Variants in *GSTP1*, which encodes an enzyme involved in detoxifying reactive oxygen species, have also been associated with asthma susceptibility.^34^ These genetic susceptibilities often follow a pattern known as the atopic march, where children with early‐onset AD are at a higher risk of developing asthma and FA later in life, reflecting a shared immunological basis.

Gene mutations play a prominent role in rare monogenic IEI, including PADs, which often share pathogenetic mechanisms with more common allergic disorders, such as epithelial barrier dysfunction and dysregulated T2 inflammation.^4^ While genetics lay the foundation for disease susceptibility, environmental factors are critical determinants of disease onset, severity, and progression. Urbanization, air pollution, secondhand tobacco smoke, dietary changes, reduced microbial exposure (hygiene hypothesis), and early antibiotic use have all been implicated in modulating immune tolerance and increasing the risk of allergic diseases.^35^, ^36^, ^37^, ^38^, ^39^ Perinatal factors such as cesarean delivery and lack of breastfeeding may impair gut microbiota development, increasing the risk of FA and AD.^40^, ^41^ Furthermore, the timing of allergen introduction appears critical; delayed exposure to common allergens such as peanuts and eggs may increase the risk of sensitization, particularly in genetically predisposed children.^42^

Ultimately, the convergence of genetic and environmental factors results in persistent immune dysregulation. Barrier dysfunction in the skin, lungs, or gut increases allergen permeability, prompting epithelial cells to release alarmins, which activate dendritic cells and promote Th2 responses. This creates a self‐reinforcing loop between inflammation and barrier damage, underpinning the chronic nature of allergic diseases. Understanding these shared mechanisms not only underscores the interconnected pathophysiology of asthma, AD, and FA but also opens avenues for early intervention. Therapeutic strategies targeting barrier repair, immune modulation, and environmental management hold promise for preventing or mitigating the burden of these increasingly common conditions.

## 4 EPITHELIAL BARRIER THEORY

The etiology of allergic diseases and immunodeficiencies is closely related to defects in the epithelial barrier, which is the first line of defense against external agents that maintain internal homeostasis. The epithelial barrier theory has been proposed to explain the significant rise in these disorders and posits that environmental toxic substances linked to industrialization, urbanization, and modern life induce epithelial barrier dysfunction. Barrier dysfunction coexists with microbial dysbiosis, characterized by decreased commensals, colonization by opportunistic pathogens, bacterial translocation to the inter‐ and subepithelial areas, tissue and circulatory inflammation, and immune dysregulation. The combination of these factors has been linked to the increasing prevalence of allergic, autoimmune, neuroimmune, and other chronic diseases and their exacerbations.^43^, ^44^ Even in IEI, a dysfunctional barrier is often the spark that ignites systemic immune dysregulation. In many IEI, genetic defects disrupt this sensing or the subsequent production of antimicrobial peptides and alarmins. PADs perfectly illustrate the epithelial‐first hypothesis. Mutations affecting *FLG* or epithelial signaling can lead to systemic immune sensitization, where a dysfunctional barrier allows allergens to trigger exaggerated Th2 responses. In IEIs affecting the intestinal mucosa (e.g., TTC7A deficiency or certain forms of very early‐onset inflammatory bowel disease [IBD]), the loss of epithelial polarity and integrity leads to a breakdown of the host‐microbiota relationship. Because of epithelial barrier damage, the microbiota that should float above the epithelium translocates between and beneath the epithelial cells. This influx of commensal bacteria into the lamina propria creates a cycle of persistent, systemic inflammation that is difficult to manage with traditional immunosuppression alone.^43^, ^44^ Therefore, IEI are a good model for understanding the complex mechanism of epithelial barrier dysfunction.

Several factors can cause epithelial barrier dysfunction, including microbiome dysbiosis, genetic and epigenetic alterations, and the exposome. The exposome has been transformed over the last several decades by industrialization, urbanization, western lifestyles, and technological advances, which have increased the frequency and forms of interactions between epithelial barriers and various environmental toxic substances that damage the epithelial barriers of the skin, respiratory tract, and gastrointestinal tract. This interaction triggers various pathological events, including epithelial cell death, epithelitis, microbial dysbiosis, pathogen colonization, and defective epithelial barrier healing.^45^, ^46^

Various models have shown that the epithelial barrier is significantly altered by external stimuli, including air pollutants, environmental factors, and other chemicals. Notably, some of these agents are present in everyday products such as toothpaste, shampoo, household laundry detergents, dishwasher rinse aid, and ultra‐processed foods that contain food emulsifiers.^47^, ^48^, ^49^, ^50^ In addition, aerosols, both anthropogenic (traffic, industry, construction, housing, agriculture, energy sector) and natural (dust from deserts, sea salt, wildfires), with varying sizes and compositions (air pollutants, pollen, dust mites, fungal spores, diesel exhaust particles, volatile organic compounds, micro‐ and nanoplastics, and ozone), can also affect the integrity of the epithelial barrier.^47^, ^48^

Interactions among different stimuli trigger the release of alarmins, which stimulate DCs, Th2 cells, or ILC2 cells, thereby increasing the production of T2 cytokines. These cytokines attract other cells, such as eosinophils and mast cells, enhance IgE class‐switching in B cells, and influence pathomechanisms, including mucus production and muscle contractility. A better understanding of the role of epithelial barrier impairment in allergic disorders and IEI is crucial for enabling personalized medicine, reliably identifying specific endotypes and biomarkers to improve the diagnosis of these reactions, and developing new therapeutic strategies to repair the epithelial barrier.

## 5 MICROBIOTA AND IMMUNE INTERACTIONS IN ALLERGY AND IMMUNE‐MEDIATED DISEASES

In allergic diseases, dysbiosis is linked to reduced levels of protective metabolites (e.g., short‐chain fatty acids [SCFAs]) and increased pro‐inflammatory signals (e.g., succinate, translocated microbe‐associated molecular patterns [MAMPs]), compromising epithelial barriers and skewing immunity toward T2 responses (ILC2/Th2 activation, IgE class‐switching, and mast‐cell priming). Rebuilding a favorable metabolite *milieu* (e.g., via fermentable fiber to boost SCFAs, targeted probiotics or defined postbiotics, and modulation of bile acid and tryptophan pathways) can enhance T regulatory (Treg) function and barrier integrity while attenuating allergic inflammation.^51^ The mucosal immune system senses MAMPs through pattern‐recognition receptors, such as toll‐like receptors, NOD‐like receptors, and others, enabling the host to interpret signals from the gut microbiota.^51^ During commensal colonization, these pathways foster immune tolerance and prevent exaggerated responses to later microbial encounters. In parallel, the intestinal microbiota shapes systemic immunity via its metabolites. Microbes produce unique small molecules that enter the circulation and are detected by receptors on epithelial, stromal, and immune cells, thereby establishing tissue‐specific immune set points across the body.^52^, ^53^

Allergic diseases arise when the immune system mounts T2 responses to otherwise innocuous antigens rather than maintaining tolerance.^54^ Perturbations in the microbiome, its localization, taxonomic composition, and metabolite output are repeatedly linked to loss of immune homeostasis and increased risk of atopic disease.^54^ Early life is a critical window. Gut dysbiosis in infancy skews T2 immunity and increases FA risk, with lower *Bifidobacteria* or reduced butyrate at 1 year predicting allergen sensitization, and decreased *Anaerostipes hadrus* and *Blautia wexlerae* marking delayed microbiome maturation and FA at 5 years.^55^, ^56^, ^57^ Expansion of *Ruminococcus gnavus*, which produces a proinflammatory polysaccharide and trace amines (tyramine, tryptamine, phenylethylamine), is associated with T2‐skewing in FA; high infant trace amines track with atopy and aberrant colonization by *Tyzzerella nexilis*, *Enterococcus faecalis*, and *R. gnavus*.^55^, ^56^, ^58^

Asthma illustrates the link between the microbiome and endotypes.^59^ Besides T2‐high disease (common in childhood), T2‐low and non–type‐2 asthma are dominated by neutrophilic inflammation.^50^, ^59^, ^60^ In adults with severe neutrophilic asthma, sputum is enriched for *Moraxella catarrhalis* and *Haemophilus influenzae*; the latter can drive Th17 responses, providing a mechanistic route to neutrophilia.^61^, ^62^, ^63^ *M. catarrhalis* similarly promotes neutrophilic inflammation in chronic obstructive pulmonary disease (COPD).^64^, ^65^ Additional taxa, including *Streptococcus pseudopneumoniae* and an unclassified *Actinobacillus*, are associated with severe neutrophilic asthma.^46^ Beyond the airway, gut features correlate with asthma severity, including reduced *Akkermansia muciniphila* and increased histamine‐producing *Morganella morganii*.^66^, ^67^

In AD, the skin microbiome is frequently enriched for *Staphylococcus aureus* and/or *S. epidermidis*, and this enrichment sometimes precedes clinical onset.^68^ Severe AD often features clonal *S. aureus* strains that elicit epidermal thickening and cutaneous Th2/Th17 responses, likely via enterotoxin A and leucocidins.^69^ Although *S. epidermidis* can fortify the barrier, overabundance promotes keratinocyte inflammation, supports *S. aureus* via biofilms, and serves as a reservoir of mobile elements conferring antibiotic resistance and virulence; in other contexts, it can limit *S. aureus* uptake by keratinocytes.^70^

Mechanistically, microbe‐derived metabolites are active in a range of allergic disorders. In allergic asthma, SCFAs, indole derivatives, polyamines, L‐tyrosine–derived products, and histamine attenuate lung inflammation by reprogramming immune‐cell function. In AD, SCFAs and indoles worsen disease severity by modulating IgE and TSLP; in FA, SCFAs, indoles, and bile acid derivatives influence pathogenesis by altering lymphocyte differentiation and related pathways. Evidence for sphingolipids remains mixed across clinical studies.^51^, ^53^

Metabolomics and immune metabolism of host cells and the microbiome are the most promising frontiers in personalized medicine, transforming the understanding of allergic conditions.^71^ It bridges the gap between complex biological data, a mechanistic understanding of type 2 inflammation, and clinical applications.^72^, ^73^ It offers a holistic view of gene–environment interactions, making it an essential pillar for the future of allergy and immunology prevention and management.^74^

## 6 OMICS TECHNOLOGIES IN ALLERGY AND CLINICAL IMMUNOLOGY RESEARCH

High‐throughput omics technologies have become valuable tools for profiling these conditions, because multiple mechanisms are involved in the development of allergic diseases. Techniques such as genomics, epigenomics, microbiomics, transcriptomics, proteomics, metabolomics, lipidomics, and glycomics enable the analysis of large biological datasets.^75^ This underpins the discovery of the molecular mechanisms underlying the development of allergic disease.^76^

Allergic diseases manifest in various forms and can occur with or without comorbidities. These conditions present diverse symptoms and underlying molecular responses. However, omics studies have uncovered several common mechanisms and cellular states across these diseases.^77^ Cytokine expression shows changes at both the transcriptomic and proteomic levels. Specifically, Th2 cytokines increase, while regulatory cytokines (IL‐10, TGF‐β) decrease. The link between these changes and allergic diseases is further supported by the restoration of normal levels after treatment with allergen immunotherapy (AIT) or monoclonal antibodies (mAbs).^78^

Omics approaches are useful not only for dissecting single allergic conditions but also for defining biomarkers that recur across diseases. A shared T2 inflammatory program is evident in asthma, AD, AR, and PAD.^79^ In the context of rare allergic diseases such as PAD, a multi‐omic approach is not merely beneficial but also essential for advancing precision medicine and transforming a scarcity of clinical data into a wealth of molecular insights. Interrogating co‐existing conditions helps separate shared from disease‐specific biology. In children, untargeted metabolomics distinguished FA from asthma by lower sphingolipid/ceramide levels in FA, consistent with influences from the microbiota, dietary restriction, and immune responses. Longitudinal multi‐omics further stratified AD into endotypes with distinct trajectories: an early‐onset persistent form (heightened T2 responses; fecal enrichment of *Ruminococcus gnavus*) and a late‐onset form (Th17 skewing; barrier defects involving CLDN2/CLDN12).^80^

Omics readouts also inform the understanding of acute, multi‐trigger syndromes such as anaphylaxis. Serum metabolomics at presentation resolves trigger‐specific fingerprints (food vs. drug) and reveals severity‐dependent metabolic shifts that persist for months after the reaction.^81^ Comparative analyses refine specificity across distinct diseases. Metabolomics distinguishes asthma, COPD, and asthma‐COPD overlap (ACO), with ACO showing a unique panel of 12 altered intermediates spanning lipid and amino acid/energy pathways.^82^ Plasma metabolomics identifies selective lipid and amino acid metabolites that discriminate between atopic and nonatopic children with asthma and healthy children, suggesting underlying mechanisms such as modulation of host–pathogen and gut microbiota interactions.^83^

Clinical translation of omics biomarkers in allergy and immunology is limited by pre‐analytical variability, small, heterogeneous cohorts, medication/diet confounding, batch and cross‐platform effects, model overfitting with weak external validation, uncertain causality, and regulatory/implementation hurdles. Addressing these demands requires harmonized protocols, multicenter longitudinal cohorts, transparent and reproducible pipelines, and prospective trials demonstrating clinical utility.^77^

The multi‐omics approach can generate biologically and clinically meaningful data, even in numerically limited cohorts, making it an ideal strategy for studying rare diseases, particularly for IEI mapping, molecular pathways, and immune effector mechanisms. For example, a multi‐omics approach has shed new light on the pathophysiology of gut inflammation in partial RAG deficiency.^84^ Therefore, it is a powerful tool for studying PAD, bridging the gap between rare and common cases by validating the functional impact of new variants and unveiling potential therapeutic targets.^85^ While more studies are integrating omics technologies into their workflows, fully understanding the biological picture requires combining multiple data layers, including omics and biological data. Although machine learning (ML)‐based approaches for omics integration are being employed, they face limitations, especially when dealing with incomplete, noisy, or out‐of‐distribution data, such as new cases that significantly differ from previous observations. In this context, incorporating deep learning (DL) and artificial intelligence (AI) tools could greatly enhance these methods by improving interpretation and elucidating underlying mechanisms.^86^ Achieving this advancement is a crucial step toward translating current knowledge into practical clinical applications, which remains an ongoing process.

## 7 BIOMARKERS FOR DISEASE PREDICTION AND PROGRESSION

Recent advances in molecular immunology have expanded the biomarker landscape for early detection and prediction, including genetic variants such as *FLG* variants. AD exhibits substantial phenotypic and endotype heterogeneity. The extrinsic phenotype (80% of cases) is characterized by elevated serum IgE levels, *FLG* variants, and a predominant T2 immune response. In contrast, the intrinsic phenotype (20%) presents with normal IgE levels, increased involvement of the Th1, Th17, and Th22 pathways, and greater resistance to therapies that typically target the T2 axis.^87^ To date, none of these candidate biomarkers has been validated and officially accepted by regulatory organizations for use in routine clinical practice and the management of AD. Elevated serum levels of periostin and DPP‐4 have been identified as significant markers for predicting favorable responses to anti‐IL‐13 therapies, such as tralokinumab.^88^ The chemokine with the greatest evidence‐based support as a potential AD biomarker, at both baseline and following therapy, is CCL17/TARC, a chemoattractant for Th2 cells that shows a robust correlation with clinical severity in both children and adults. In respiratory allergy, biomarkers are now central to patient stratification and therapeutic decision‐making.^89^ In asthma, eosinophil count, fractional exhaled nitric oxide (FeNO), and periostin are used to identify T2‐high endotypes that are responsive to T2‐targeted biologic therapies.^90^ Component‐resolved diagnostics (CRD) enable the identification of specific allergen sensitivities, improve diagnostic accuracy, and guide decisions on allergen immunotherapy.^91^ CRD, and epitope‐level assays, such as bead‐based epitope assays (BBEA), are also improving the prediction of clinical reactivity. For example, sIgE to Ara h 2 peptide correlates more strongly with true peanut allergy and the risk of systemic reaction than traditional whole‐extract sIgE.^91^ Beyond IgE, functional cellular biomarkers are gaining traction as well. The basophil activation test (BAT) shows excellent specificity for diagnosing IgE‐mediated allergies, with high sensitivity for peanut, milk, and egg allergies, especially in patients with borderline sIgE or an equivocal clinical history, and is also used to predict reaction severity and to monitor oral immunotherapy (OIT).^92^

A growing number of IEI is now recognized as presenting with prominent allergic phenotypes. Biomarkers play a central role in distinguishing these rare syndromes. For instance, high levels of T2‐associated biomarkers, including markedly elevated total IgE, severe blood eosinophilia, elevated serum tryptase, and reduced Th17 and regulatory T cell counts, are key laboratory parameters that support the diagnosis and stratification of PAD. The genetic and immunologic biomarkers not only enable early and precise diagnosis but also guide treatment choices. Epigenetic markers, specifically DNA methylation patterns, have emerged as stable tools for immune monitoring. Unlike RNA, which can be highly volatile, DNA methylation provides a consistent record of cell lineage and activation states.

Drug hypersensitivity is a complex field that requires biomarkers to navigate diverse phenotypes, ranging from IgE‐mediated anaphylaxis to T‐cell‐driven delayed hypersensitivity. In delayed‐type drug hypersensitivity, T cell–based functional assays, such as the lymphocyte transformation test (LTT), detect drug‐specific memory T cells by measuring their proliferation. These tools are increasingly used to identify the cellular mechanisms behind type IV hypersensitivity and to guide safe drug reintroduction.^93^ The Mas‐related G‐protein‐coupled receptor member X2 (MRGPRX2), predominantly expressed on mast cells, is also under investigation. It mediates IgE‐independent immediate drug hypersensitivity reactions by directly activating cationic drugs, such as neuromuscular blockers. No validated in vivo or in vitro biomarkers exist for MRGPRX2‐mediated reactions. Functional assays, such as the direct mast cell activation test (MAT), MRGPRX2‐silenced passive MAT, and conditioned basophil testing, are being investigated to characterize this endotype.^94^ Additionally, MRGPRX2 polymorphisms are emerging as candidate genetic biomarkers because of their potential influence on receptor responsiveness. At the same time, specific HLA alleles (e.g., HLA‐B57:01 and HLA‐B15:02) are associated with different drug hypersensitivity reactions.^94^ Real‐world examples, such as HLA‐B57:01 screening to prevent abacavir reactions, illustrate how biomarkers are already transforming the care of patients with allergic diseases. Ultimately, they are key enablers of personalized medicine in clinical immunology.

Looking forward, this field is rapidly evolving. Novel technologies, including high‐dimensional mass cytometry, transcriptomics, and epigenetic profiling, are expanding the roster of potential biomarkers. The hope is that some of these cutting‐edge markers will prove reliable and convenient enough for clinical use. Precision medicine in allergy/immunology will likely involve a combination of established and emerging biomarkers: well‐established ones (such as IgE levels or genetic tests) alongside newer tools (such as cell activation profiles or molecular “omics” signatures).^77^ As these are validated, clinical guidelines will incorporate them for risk prediction, disease monitoring, and therapy selection. The ultimate vision is that, by understanding an individual patient's unique biomarker profile, we can predict disease before it strikes, prevent or mitigate its course through early interventions, and personalize treatments to achieve the best outcomes with minimal side effects. Achieving this vision will require continued research and collaboration, but progress to date has already demonstrated the lifesaving impact of biomarkers in our field. Biomarkers for disease prediction and progression are not only deepening our understanding of pathogenesis but also provide tangible tools that bring us closer to truly personalized medicine in allergology and clinical immunology.

## 8 PRECISION MEDICINE: THE PARADIGM OF CVID

Precision medicine in clinical immunology represents the pinnacle of bench‐to‐bedside translation. It is defined as the transition from managing symptoms and providing general immune support to delivering the right treatment for the right patient at the right time, based on the specific molecular pathophysiology of their disease. In the context of immunology, precision medicine is centered on identifying the molecular defects that drive immune dysregulation and translating these insights into targeted therapies. Rather than broad immunosuppression, this approach targets signaling pathways responsible for disease pathogenesis.^95^

The heterogeneous group of CVID conditions is a clear example of how precision medicine tools have revolutionized diagnostic and therapeutic approaches in clinical immunology. In the context of CVID, precision medicine is about unmasking the individual within the cohort. By moving away from a one‐size‐fits‐all diagnosis and adopting deep molecular phenotyping, its variable nature can finally be addressed, providing patients with a therapeutic roadmap as unique as their own immune system. CVID has historically been a diagnosis of exclusion, defined operationally by hypogammaglobulinemia, impaired specific antibody production, and the absence of other known immunodeficiencies. This view is now obsolete, as advances in genomics have revealed that the CVID clinical syndrome is a common endpoint for a heterogeneous collection of IEI. The identification of monogenic defects in 10–50% of CVID cohorts is revolutionizing patient care, transforming a broad clinical diagnosis into a landscape of precise, actionable molecular defects.^95^ The profound clinical variability of CVID is best understood by directly linking its clinical endotypes—infection, autoimmunity, and lymphoproliferation/malignancy—to their underlying genetic drivers. This genotype–phenotype integration provides a powerful tool for prognostication and guides patient‐specific monitoring.

The most severe endotypes, characterized by aggressive, early‐onset autoimmunity and lymphoproliferation, are frequently driven by defects in critical T‐cell regulatory pathways. For example, heterozygous mutations in *CTLA4* or biallelic mutations in its trafficking partner *LRBA* cripple a key immune checkpoint, leading to unchecked T‐cell activation.^96^, ^97^ The clinical consequence is a devastating phenotype of autoimmune cytopenias, severe enteropathy, and lymphocytic infiltration of organs, including the lungs (granulomatous lymphocytic interstitial lung disease). Similarly, pronounced lymphoproliferation, leading to splenomegaly, a high risk of B‐cell lymphoma, and progressive bronchiectasis, is the hallmark of Activated PI3K‐Delta Syndrome (APDS), which arises from gain‐of‐function (GOF) mutations in *PIK3CD* or LOF mutations in its regulatory subunit *PIK3R1*, causing constitutive activation of the PI3Kδ signaling pathway.^98^, ^99^

However, other genetic defects have different risk profiles. Pathogenic variants in *NFKB1*, now recognized as a frequent monogenic cause of CVID, often result in late‐onset disease. While patients have a clear antibody deficiency, the penetrance of severe immune dysregulation may be lower than that of defects in the CTLA‐4 or PI3K pathways. A genetic diagnosis is therefore paramount; it reframes a patient's prognosis from a statistical probability within a heterogeneous CVID cohort to a predictable trajectory based on a defined molecular lesion, mandating specific treatment and surveillance strategies for complications such as lymphoproliferation, organ‐specific autoimmunity, and cytopenias.

## 9 PRECISION MEDICINE IN CLINICAL IMMUNOLOGY: HARNESSING MECHANISMS OF IMMUNE (DYS)REGULATION

The molecular elucidation of CVID has enabled a strategic therapeutic shift, moving beyond immunoglobulin replacement therapy IgRT and broad immunosuppression toward mechanism‐based interventions. These interventions can be classified into two main categories: adapted and repurposed immunomodulators and novel, purpose‐built targeted therapies. This approach leverages existing drugs, originally approved for other indications, such as rheumatologic diseases or cancer, that fortuitously and preferentially target the specific biological pathways disrupted in certain IEI. An excellent example of this approach is the use of abatacept. Initially developed for rheumatoid arthritis, abatacept is a CTLA4‐Ig fusion protein. It has been successfully repurposed to treat defects in the CTLA‐4 pathway. By acting as a soluble surrogate for the deficient or dysfunctional CTLA‐4 protein, abatacept restores inhibitory signaling to T cells, leading to remarkable control of severe autoimmune and inflammatory manifestations in patients with *CTLA‐4* and *LRBA* mutations.^100^ Another example is JAKi, such as ruxolitinib and tofacitinib, which were originally developed to treat myeloproliferative neoplasms and autoimmune disorders. They have since been used, with mixed results, to treat predominantly immunodysregulatory manifestations of *STAT1‐* or *STAT3‐*GOF‐associated disease, which can present with hypogammaglobulinemia and severe autoimmunity/autoinflammation.^101^ Sirolimus and other mTOR inhibitors are established immunosuppressants that inhibit the mTOR pathway, a central hub for cell growth and proliferation. Because mTOR is downstream of PI3K, sirolimus has been used to treat dysregulated lymphocyte proliferation in APDS and related disorders, with clinical efficacy in reducing lymphadenopathy and splenomegaly.

The cutting edge of precision medicine is represented by drugs designed de novo to correct the specific molecular defect underlying an IEI. While idelalisib represents the first PI3Kδ inhibitor originally developed and used for relapsed chronic lymphocytic leukemia,^102^ leniolisib is the prime example of a purpose‐built therapy in IEI. It is a selective small‐molecule inhibitor of the p110δ catalytic subunit of PI3K that ameliorates immune dysbalance by normalizing lymphocyte proliferation and function. It was specifically developed to treat APDS by directly blocking the hyperactive enzyme responsible for the disease. In a landmark randomized controlled trial, leniolisib was shown to normalize immune dysregulation, reduce lymph node and spleen size, and improve the immunophenotype of patients with APDS, leading to its approval as the first targeted treatment for this condition.^103^ Its high selectivity for the delta isoform enables more precise intervention, potentially with fewer off‐target effects than broader inhibitors such as sirolimus. Indeed, in patients with activating *PIK3CD* mutations causing APDS, hyperactive PI3Kδ signaling disrupts lymphocyte development, producing combined immunodeficiency with autoimmunity, atopy, and lymphoproliferation. Atopy here arises from excess PI3K–AKT–mTOR activity, skewing CD4^+^ T cells toward Th2, compromising Tregs, and distorting B‐cell class switching.^104^

In addition to CVID, other rare monogenic IEI, particularly those with prominent immune dysregulation, offer powerful windows into the pathways that underlie more common allergic and autoimmune diseases.^90^ Discrete molecular defects in Tregs, cytokine signaling, or PI3Kδ‐mediated lymphocyte development dismantle immune tolerance, creating opportunities for targeted therapy.^4^, ^105^

Tregs maintain peripheral tolerance; when they fail, unchecked effector activity can drive severe allergy and autoimmunity. *FOXP3* mutations in IPEX syndrome represent the classic prototype of Treg failure. Yet other mechanisms converge on the same outcome: *IL2‐receptor α* (*IL2RA*) and *STAT5B* mutations impair IL‐2–STAT5 signaling, blocking IL‐10 production and destabilizing Tregs. DOCK8 deficiency compromises IL‐2–STAT5 activation, further reducing Treg survival while skewing CD4^+^ cells toward Th2 responses. Even structural defects, as in WAS, impair TCR signaling and cytoskeletal remodeling, weakening Treg, B cell, and dendritic cell function. Collectively, these examples underscore that diverse molecular defects culminate in Treg dysfunction and lead to the collapse of immune tolerance.^95^

Many of these T2‐associated cytokine effects converge on the JAK–STAT pathway, and mutations such as *STAT3*‐LOF, *STAT3*‐GOF, *STAT1*‐GOF, *JAK1*‐GOF, and *STAT6*‐GOF exaggerate T2 responses or weaken tolerance.^95^ Some disorders, however, extend beyond cytokine dysregulation.^105^

**FIGURE 1:**
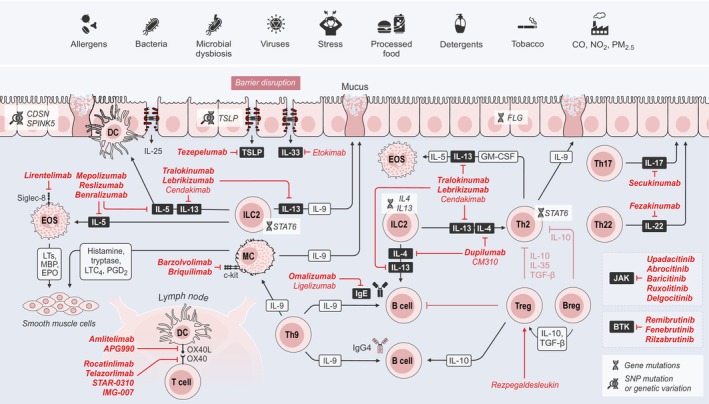
An overview of T2 inflammatory mechanisms with their main key drivers and pharmacological blockers.

Small‐molecule inhibitors, including JAKi (baricitinib, upadacitinib, ruxolitinib), interrupt JAK1/2‐mediated cytokine signaling, including Th2 cytokines (Table 1, Figure 1). They provide rapid relief in AD and are under investigation for broader immune dysregulation syndromes. Concerns remain about the possible risks, particularly if used long‐term.^10^, ^105^

**TABLE 1:** Summary of the main reports of targeted therapies in IEI with atopic phenotype.^10^, ^129^

Targeted therapy	PAD	Main indications
Dupilumab	STAT3 AD‐HIES (Job syndrome)	Skin manifestations, pruritus, IgE reduction, EoE
	DOCK8 AR‐HIES	Skin manifestations as bridge to HSCT
	ZNF341 deficiency	Severe atopic dermatitis, IgE reduction
	*STAT6* GOF	Allergic dysregulation, atopic dermatitis, and asthma
	SPINK5 deficiency (Netherton syndrome)	Skin manifestations
	WAS	Atopic dermatitis
	ARPC1B deficiency	Severe atopic dermatitis, vasculitis, and colitisrefractory to conventional medical therapy
	CADINS (CARD11 deficiency)	Allergic dysregulation
	Omenn Syndrome	Bridge to HSCT
	IPEX and IPEX‐like syndrome	Skin manifestations (atopic dermatitis)
Omalizumab	STAT3 AD‐HIES (Job syndrome)	Cutaneous and pulmonary manifestations, IgE reduction
	DOCK8 AR‐HIES	Skin manifestations as bridge to HSCT
	SPINK5 deficiency (Netherton syndrome)	Skin manifestations
	WAS	Diffuse pruritic atopic dermatitis resistant to conventional systemic immunosuppressive therapy
Mepolizumab	CADINS (CARD11 deficiency)	Allergic dysregulation
	STAT3 AD‐HIES (Job syndrome)	Atopic dermatitis and other allergic manifestations
Reslizumab	STAT3 AD‐HIES (Job syndrome)	Atopic dermatitis and other allergic manifestations
Benralizumab	STAT3 AD‐HIES (Job syndrome)	Atopic dermatitis and other allergic manifestations
Kallikrein inhibitors	SPINK5 deficiency (Netherton syndrome)	/
MALT1 inhibitors	CBM‐opathies	/
Ruxolitinib	IL6ST deficiency	Autoinflammation and immune dysregulation manifestations
	*STAT6* GOF	Allergic dysregulation, skin disease/atopic dermatitis
	*STAT1* GOF	Autoimmune and autoinflammatory manifestations
Tofacitinib	IL6ST deficiency	Autoinflammation and immune dysregulation manifestations
	*STAT6* GOF	Allergic dysregulation, skin disease/atopic dermatitis
Baricitinib	*JAK1* GOF	Atopic dermatitis, chronic inflammatory arthritis, or inflammatory bowel diseases
	*STAT1* GOF	Autoimmune and autoinflammatory manifestations
Upadacitinib	JAK1 GOF	Atopic dermatitis, chronic inflammatory rheumatism, or inflammatory bowel diseases
Leniolisib	APDS	Significant infectious and/or immunodysregulatory phenotype
Abatacept	CTLA4 deficiency	Immune dysregulation: autoimmune and lymphoproliferative manifestations
Ustekinumab	SPINK5 deficiency (Netherton syndrome)	/
	*CARD14* GOF	CARD14‐associated papulosquamous eruption, namely psoriasis and pityriasis rubra pilaris
	*DSG1* LOF	/
Ixekizumab, secukinumab	SPINK5 deficiency (Netherton syndrome)	/
	*DSG1* LOF	/
Anti‐TNF agents	SPINK5 deficiency (Netherton syndrome)	/
Anakinra	WAS	Autoinflammatory manifestations
Candidate PLCG2 inhibitors	PLAID	/

Abbreviations: AD‐HIES, autosomal dominant hyper‐IgE syndrome; APDS, activated phosphoinositide 3‐kinase delta syndrome; AR‐HIES, autosomal recessive hyper‐IgE syndrome; ARPC1B, actin‐related protein 2/3 complex subunit 1B; BCL10, B‐Cell lymphoma/leukemia 10; CADINS, CARD11‐associated atopy with dominant interference of NF‐κB signaling; CARD, caspase recruitment domain family member; CBM‐opathies, CARD11–BCL10–MALT1 complex diseases; CTLA4, cytotoxic T‐lymphocyte–associated protein 4; DOCK8, dedicator of cytokinesis 8; DSG1, desmoglein 1; EoE, eosinophilic esophagitis; GOF, gain Of function; HSCT, hematopoietic stem cell transplantation; IgE, immunoglobulin E; IL6ST, interleukin 6 signal transducer; IPEX, immune dysregulation, polyendocrinopathy, enteropathy, X‐linked; JAK1, Janus kinase 1; LOF, loss of function; MALT1, mucosa‐associated lymphoid tissue lymphoma translocation protein 1; PAD, primary atopic disorders; PLAID, PLCG2‐associated antibody deficiency and immune dysregulation; PLCG2, phospholipase C gamma 2; SPINK5, serine peptidase inhibitor, Kazal type 5; STAT, signal transducer and activator of transcription; TNF, tumor necrosis factor; WAS, Wiskott–Aldrich syndrome; ZNF341, zinc finger protein 341.

Immune dysregulation is not random but mechanistically patterned. Precision immunology increasingly relies on biomarker‐guided patient stratification. In the future, integration of genetic screening with immune profiling and, potentially, lesional transcriptomics will further refine therapy selection, ensuring that interventions match the underlying molecular defect.^10^

## 10 MONOCLONAL ANTIBODIES AND CYTOKINE INHIBITORS IN ALLERGY

If most allergic patients respond well to conventional treatment strategies, a small percentage have severe, difficult‐to‐treat disease that remains poorly controlled, unresponsive, or dependent on high‐dose conventional treatments, including systemic corticosteroids or immunosuppressants. The consequences include high healthcare utilization, such as hospital admissions, school or work absenteeism, and treatment side effects.^20^ The progressive understanding of the complex pathogenesis of the allergic cascade and inflammatory pathways has enabled the identification of specific therapeutic targets, revolutionizing the treatment of allergic diseases.

Therapeutic strategies, including humanized mAbs and small‐molecule JAKi, target IgE, T2 cytokines or receptors, and alarmins, or inhibit signaling by a broad range of inflammatory cytokines.^106^, ^107^ These agents play a central role in controlling allergic diseases and provide clinically significant reductions in disease burden. Their indications as adjunctive treatments are expanding for pediatric allergic conditions inadequately controlled by conventional strategies, including asthma, chronic spontaneous urticaria (CSU), AD, and EoE (Tables 2, 3, 4, respectively).^25^, ^106^, ^108^, ^109^, ^110^, ^111^ These treatments have a globally acceptable adverse‐event profile.^106^, ^107^ They may also positively affect innate immunity, such as improving antiviral responses, as seen with omalizumab.^112^ However, they can also interfere with non‐T2 inflammatory epithelial pathways, as reported with mepolizumab, thereby increasing the risk of asthma exacerbations.^113^ These treatments enable a ‘treat to target’ strategy and a personalized approach. In cases of multimorbidity, some of them can be used to treat multiple allergic diseases.

**TABLE 2:** Monoclonal antibodies and small molecules approved for allergic diseases in children.

Biological agent	Molecular target	Indication/Disease	Age range of approval
Omalizumab	IgE	Moderate^a^ to severe^a^, ^b^ allergic asthma	≥6 years
		CSU^a^, ^b^	≥12 years
		FA^a^	≥1 year
Dupilumab	IL‐4Ra	Moderate^a^ to severe^a^, ^b^ eosinophilic^a^/T2^b^ asthma	≥6 years
		CRSwNP^a^, ^b^	≥12 years^a^; ≥ 18 years^b^
		Moderate to severe AD^a^, ^b^	≥6 months
		CSU^a^	≥12 years
		EoE^a^, ^b^	≥1 year (at least 15 kg)
		AFRS^a^	≥12 years
Tralokinumab	IL‐13	Moderate to severe AD^a^, ^b^	≥12 years
Lebrikizumab	IL‐13	Moderate to severe AD^a^, ^b^	≥12 years
Mepolizumab	IL‐5	Severe eosinophilic asthma^a^, ^b^	≥6 years
		EGPA^a^, ^b^	≥18 years^a^; ≥ 6 years^b^
		HES^a^, ^b^	≥12 years^a^; ≥ 18 years^b^
Benralizumab	IL‐5R	Severe eosinophilic asthma^a^, ^b^	≥6 years^a^
			≥18 years^b^
Tezepelumab	TSLP	Severe asthma^a^, ^b^ irrespective of biomarker levels	≥12 years
		CRSwNP^a^, ^b^	≥12 years^a^; ≥ 18 years^b^
Upadacitinib	JAK	Refractory^a^, moderate‐to‐severe AD^a^, ^b^	≥12 years
Abrocitinib	JAK	Refractory^a^, moderate‐to‐severe AD^a^, ^b^	≥12 years
Baricitinib	JAK	Moderate‐to‐severe AD^b^	≥2 years

Abbreviations: AD, atopic dermatitis; AFRS, allergic fungal rhinosinusitis; CRSwNP, chronic rhinosinusitis with nasal polyps; CSU, chronic spontaneous urticaria; EGPA, eosinophilic granulomatosis with polyangiitis; EoE, eosinophilic esophagitis; FA, food allergy; HES, hypereosinophilic syndrome; IL, interleukin; JAK, Janus Kinase; TSLP, thymic stromal lymphopoietin.

^a^ FDA approval.

^b^ EMA approval.

**TABLE 3:** Available biological therapies in pediatric moderate (FDA) to severe (EMA) asthma.

Biologic agent	Phenotype of asthma	Age	Biomarkers	Predictors of good response
Omalizumab^a^, ^b^	Allergic asthma	≥6 years	Total serum IgE level: 6–11 years: 30–1500 IU/mL ≥12 years: 30–700 IU/mL Evidence of sensitization to at least one perennial aeroallergen, regardless of eosinophil count	Blood eosinophilia >250 μ/L FeNO ≥20 ppb Allergen‐driven symptoms Childhood‐onset asthma
Mepolizumab^a^, ^b^	Eosinophilic asthma	≥6 years	Eosinophil count ≥150 cells/μL	Blood eosinophilia Exacerbations in the previous year Nasal polyposis Late‐onset asthma
Benralizumab^a^, ^b^	Eosinophilic asthma	≥6 years^a^ ≥18 years^b^	Eosinophil count ≥300 cells/μL	Blood eosinophilia
Dupilumab^a^, ^b^	Eosinophilic^a^/T2^b^ asthma	≥6 years	Eosinophil count ≥150 cells/μL IgE ≥30 IU/mL FeNO ≥20 ppb	Blood eosinophilia ≥150 cells/μL FeNO ≥20 ppb (6–11 years) FeNO≥25 ppb (12+ years) Presence of other allergic comorbidities
Tezepelumab^a^, ^b^	Any phenotype of asthma	≥12 years	Not applicable	Not applicable

Abbreviation: FeNO, fractional exhaled nitric oxide.

^a^ FDA approval.

^b^ EMA approval.

**TABLE 4:** Newly approved biological therapies for moderate‐to‐severe atopic dermatitis not adequately controlled with topical treatments.

Biologic agent	Molecular target	Age	Indications	Comments
Dupilumab	IL‐4/IL‐13	≥6 months^a^, ^b^	Moderate‐to‐severe AD	High efficacy for inflammation and itch Beneficial for other T2‐mediated diseases; marked reductions in IgE levels
Tralokinumab	IL‐13	≥12 years^a^, ^b^	Moderate‐to‐severe AD	Moderate efficacy for inflammation and itch
Lebrikizumab	IL‐13	≥12 years^a^, ^b^	Moderate‐to‐severe AD	High efficacy for inflammation and itch
Nemolizumab	IL‐31R	≥12 years^a^, ^b^	Moderate‐to‐severe AD	High efficacy for itch
Upadacitinib	JAK1	≥12 years^a^, ^b^	Refractory^a^, moderate‐to‐severe AD	Typically, second line to biologics Not recommended in combination with other JAK inhibitors, biologic immunomodulators, or other immunosuppressants Avoid use of live vaccines immediately before, during, and immediately after treatment It may be considered for short courses and for individuals with autoimmune disorders
Abrocitinib	JAK1	≥12 years^a^, ^b^	Refractory^a^, moderate‐to‐severe AD	
Baricitinib	JAK1/‐2	≥2 years^b^	Moderate‐to‐severe AD	

Abbreviations: AD, atopic dermatitis; IL: interleukin; JAK, Janus Kinase.

^a^ FDA approval.

^b^ EMA approval.

Omalizumab is a recombinant mAb that targets the Fc fragment of IgE, thereby preventing binding to FcεRI on MCs and basophils and reducing free IgE. It was the first biological therapy approved for the treatment of moderate (FDA) to severe (FDA and EMA) allergic asthma in adults and adolescents (≥12 years).^106^, ^107^, ^108^, ^114^ Subsequently, it was licensed for the treatment of CSU and allergic asthma in children and was recently approved by the FDA for the treatment of food allergies.^106^, ^107^, ^108^, ^114^ Ligelizumab is an anti‐IgE mAb that suppresses free IgE more effectively than omalizumab. However, this increased potency has not translated into improved therapeutic efficacy, and trials have been discontinued.^106^, ^107^

Dupilumab is a fully humanized mAb that inhibits IL‐4 and IL‐13 signaling by binding to the IL‐4 receptor α (IL‐4Rα). It was first approved by the EMA for the treatment of inadequately controlled moderate‐to‐severe AD both in adults and adolescents. Dupilumab is currently indicated for the management of other T2 diseases, including T2 moderate to severe asthma, EoE, and severe AD in young children.^106^, ^107^, ^108^, ^110^, ^111^, ^112^, ^113^ Tralokinumab and lebrikizumab are two recently approved mAbs for the treatment of moderate‐to‐severe AD. Both bind soluble IL‐13, but tralokinumab also blocks IL‐13 from binding to its receptor.^106^, ^107^, ^108^ Mepolizumab and reslizumab neutralize IL‐5, preventing its interaction with the receptor and thereby reducing eosinophil production and survival. Benralizumab blocks the interaction between IL‐5 and IL‐5R by binding to the Fab domain, with a similar effect on eosinophil differentiation and maturation. Mepolizumab, reslizumab, and benralizumab have been approved for the treatment of moderate‐to‐severe eosinophilic asthma.^108^, ^114^ Mepolizumab recently received approval for the treatment of eosinophilic granulomatosis with polyangiitis (EGPA^106^, ^107^).

The epithelial‐derived cytokine TSLP is highly expressed in AD and is closely associated with disease severity and impaired skin barrier function. TSLP signals through a receptor complex that engages JAK1/JAK2, primarily activating STAT5, thereby driving DC maturation and OX40L upregulation. The interaction between OX40 and OX40L promotes T cell activation, proliferation, survival, and differentiation, enhances effector and memory T cell responses, and suppresses Treg function. Tezepelumab is a human mAb that inhibits TLSP and the interaction with its receptor. It is now indicated for the management of severe asthma (≥12 years of age) regardless of T2‐high or T2‐low phenotype.^106^, ^107^, ^108^

Given the pathogenic role of JAK/STAT signaling in T2 responses, the small‐molecule JAK inhibitors have been identified and used to treat allergic disorders.^115^ As mentioned above, immunosuppressant JAKi have recently been approved by the EMA for the management of moderate‐to‐severe AD, and trials are planned for other allergic diseases, such as FA.^106^, ^107^, ^108^ While drugs targeting the IL‐4/IL‐13 axis are mainly effective in patients with T2‐dominant inflammation, in cases with Th1/Th17/Th22 activity, which is typical for intrinsic AD, adults, or patients of Asian descent, JAKi (e.g., baricitinib and upadacitinib) demonstrate greater efficacy.^87^

In moderate‐to‐severe AD, rezpegaldesleukin is a first‐in‐class biologic (currently in a phase 2b clinical trial) that selectively targets the IL‐2R (a peg‐IL‐2R agonist), activating Tregs rather than other immune cells, potentially leading to long‐term, treatment‐free disease control.^116^ Human Treg cells exhibit phenotypic plasticity and can be differentiated into effector T cells (Th1, Th2, or Th17) in an inflammatory environment. Effector cells produce pathogenic cytokines, such as IFN‐γ, IL‐4, IL‐13, and IL‐17. The instability of Treg cells is a crucial limiting factor for the successful development of adoptive cell therapy (ACT [e.g., lentivirus‐mediated *FOXP3* gene transfer to CD4^+^ T cells]) with Treg cells in immune diseases.^117^ Targeting the T‐cell co‐stimulatory molecules represents another viable treatment option for moderate‐to‐severe AD. OX40/OX40L signaling can promote effector T cell proliferation and inhibit Treg cell function; blockade of OX40 (rocatinlimab) or OX40L (amlitelimab) inhibits the survival and activation of effector T cells generated from either naive or memory T cells, restoring Treg function.^118^, ^119^

Despite these advances, unmet needs remain, particularly for age groups (e.g., <6 years for severe asthma) and disease phenotypes/endotypes (e.g., low T2 disease). It is also essential to highlight the lack of pediatric clinical trials that consider age‐specific characteristics or outcomes, as well as the cost, which is a barrier in low‐income countries. Finally, criteria for selecting the optimal treatment, consensual core outcome measures set for effectiveness assessment, long‐term efficacy and safety data, potential for treatment discontinuation, effects on disease trajectories, and the risk of developing other allergic conditions, require further studies in children.^1^

## 11 AIT IN THE ERA OF PRECISION MEDICINE

AIT began over 100 years ago with individual and experimental approaches, primarily in patients with respiratory allergies and insect‐venom hypersensitivity.^120^ Without specific knowledge of pathophysiological mechanisms, pollen extracts were administered to allergic individuals with significant success.^121^ Over the years, individual initiatives have also explored desensitization induction toward tolerance to allergenic foods, with a small series of individuals treated with increasing amounts of food, again without standardized protocols, but with some success.^122^ Clearly, early AIT was applied in a personalized and intuitive manner, albeit without substantial scientific evidence.

With the development of molecular allergology, new tools have become available to study patient profiles, particularly their IgE sensitization profiles, which can be compared with the likelihood of AIT success. For example, pollen‐allergic patients can undergo optimized AIT after precise mapping of sensitization using IgE measurements to major pollen allergens available for therapy.^121^ For FA, IgE specific to molecules such as Ara h 2 in peanut has been linked to the outcome of oral tolerance induction.^123^ Thus, molecular allergology has clearly contributed to a more personalized approach to AIT and to the development of precision medicine.

The field of AIT has clearly evolved toward safer, higher‐quality extracts and improved patient selection. Nevertheless, real precision medicine can be achieved when a specific sensitization profile is matched to a personalized allergen extract. AIT represents a model of precision medicine, often regarded as its prototypical example owing to its capacity to customize interventions at the most individualized level.^124^ The development of AIT reflects the overall trajectory of precision medicine, evolving from empiricism based on the relief of clinical manifestations to approaches informed by pathophysiological mechanisms and toward highly granular strategies grounded in molecular and immunological insights. Unlike other therapeutic options for IgE‐mediated allergic diseases, AIT may be directed to the specific IgE profile of each patient, enabling not only targeted modulation of the immune response but also alteration of the natural history of the disease.^124^, ^125^ This disease‐modifying effect, unmatched by treatments targeting signs and symptoms, underpins AIT's unique pharmacoeconomic profile, as the durability of its benefits translates into long‐term reductions in features such as morbidity, healthcare utilization, and overall economic burden.^126^

A cornerstone of precision medicine in AIT is precision allergy molecular diagnosis, which has transformed clinical practice through component‐resolved diagnostics.^126^ By identifying IgE reactivity to individual allergen molecules rather than extracts, these techniques enable clinicians to distinguish genuine sensitization from cross‐reactivity and to define the molecular landscape of each patient's sensitization profile.^125^ This information is particularly critical in polysensitized patients, where conventional diagnostic tools may fail to provide sufficient data.^126^ In addition to improving diagnostic accuracy, molecular profiling refines risk assessment, with specific allergen components serving as biomarkers of disease severity or predictors of progression from sensitization without clinical manifestations.

Building on precision diagnostics, patient stratification represents the next critical dimension of personalized AIT. The treatable traits concept, which integrates genetic, endotypic, phenotypic, and psychosocial features, enables clinicians to identify subgroups with differential responses or risks, thereby avoiding inappropriate interventions and improving outcomes. This stratification is particularly relevant in complex respiratory disorders, where overlapping mechanisms and comorbidities necessitate individualized treatment approaches. Within the AIT, stratification informs the optimization of therapeutic parameters, including the route of administration, dosing regimens, and allergen composition. Advances in regulatory science have reinforced this precision approach by mandating stringent standardization and quality control of allergen products, ensuring consistent efficacy and safety.^124^ At the same time, the development of innovative technologies, such as recombinant allergens, hypoallergenic derivatives, and novel delivery systems, is expanding the therapeutic repertoire and further aligning AIT with the principles of precision medicine.^124^, ^125^, ^126^, ^127^

The precision framework also extends to monitoring and follow‐up, where identifying and validating biomarkers for efficacy, safety, and long‐term outcomes remains a pressing priority. Biomarkers that predict clinical benefits, confirm tolerance induction, or signal relapse after treatment discontinuation would substantially enhance the clinical utility of AIT. Parallel innovations in digital health technologies, including integrated care pathways, sentinel surveillance networks, and clinical decision support systems, are beginning to enable real‐time data integration, facilitating patient stratification, monitoring adherence, and guiding therapeutic adjustments.^124^, ^125^, ^126^, ^127^ These digital tools, combined with an increasing emphasis on shared decision‐making, reinforce the participatory dimension of precision medicine. Patient engagement in activities such as defining treatment goals, choosing routes of administration, and aligning expectations has been shown to improve adherence and satisfaction, thereby enhancing therapeutic success.

Finally, the pharmacoeconomic dimension of AIT underscores its status as an almost ideal precision‐medicine model.^124^ By targeting the right patient with the right intervention at the right time, AIT generates long‐term benefits that extend beyond controlling clinical manifestations to fundamentally modify disease trajectories. This not only has the potential to reduce direct healthcare costs but also to mitigate the indirect burden associated with chronic allergic disease, potentially positioning AIT as both clinically transformative and economically sustainable.

Taken together, the evolution of AIT from empirical practice to a science‐driven, molecularly guided, and patient‐centered intervention exemplifies the realization of precision medicine in allergy and immunology. The convergence of tools such as molecular diagnostics, multi‐omics platforms, functional assays, and digital technologies has created a framework in which AIT is no longer simply a treatment but a paradigm for tailoring medicine. Indeed, AIT not only embodies the principles of precision medicine, including being predictive, preventive, and participatory, but also provides a transformative blueprint for broader implementation, optimizing clinical outcomes, minimizing adverse events, and ultimately reshaping the natural course of allergic diseases.

## 12 CONCLUSION

The integration of precision medicine marks a definitive shift in how to approach the spectrum of pediatric allergy and immunology, bridging the gap between rare monogenic disorders and common polygenic allergic conditions. By leveraging advanced genomic sequencing, deep immunophenotyping, and “omics,” clinicians are no longer limited to treating generalized symptoms but can instead target the specific molecular drivers of a child's disease. This paradigm shift not only accelerates the diagnostic journey for rare IEI but also paves the way for personalized therapeutic interventions in widespread conditions. Precision tools allow for the identification of specific endotypes in common ailments, such as severe asthma and AD. This means moving beyond a one‐size‐fits‐all approach toward the strategic use of biologics and small molecules that target the specific inflammatory cascade active in each patient. Simultaneously, AI‐powered analytics and sophisticated big data platforms will help clinicians analyze diverse datasets, identify previously undetected disease patterns, and improve predictive models, thereby accelerating the implementation of precision medicine in pediatric practice.^128^

Furthermore, the study of rare IEI and PAD offers an invaluable window into the fundamental mechanisms of human immunity. Insights gained from monogenic defects often reveal master switches of inflammation. By understanding these extremes, more sophisticated treatments can be developed for children with common allergies, transforming chronic management into proactive, disease‐modifying care.

Shared biological pathways between rare and common diseases have deepened, and the promise of more effective, tailored, and proactive healthcare has become a reality. In the near future, pediatric allergy and immunology will be defined not only by the severity of clinical manifestations but also by the precision of their cures.

## AUTHOR CONTRIBUTIONS

**Martha Jimenez Freites:** Investigation; writing – review and editing. **Ivan Taietti:** Investigation; writing – original draft; writing – review and editing; methodology. **Mattia Giovannini:** Investigation; writing – review and editing. **Rubén Fernández‐Santamaría:** Investigation; writing – review and editing. **Aspasia Karavelia:** Investigation; writing – review and editing. **Maria Isabel Delgado‐Dolset:** Investigation; writing – review and editing. **Marketa Bloomfield:** Investigation; writing – review and editing. **Martina Votto:** Investigation; writing – original draft; methodology; writing – review and editing; data curation. **Janice Layhadi:** Investigation; writing – review and editing. **Jonathan Hourihane:** Investigation; writing – review and editing. **Cezmi A. Akdis:** Investigation; writing – review and editing. **Francesco Cinetto:** Investigation; writing – review and editing. **Urszula Radzikowska:** Investigation; writing – review and editing. **Mubeccel Akdis:** Investigation; writing – review and editing. **Riccardo Castagnoli:** Conceptualization; investigation; writing – original draft; writing – review and editing; methodology; data curation; supervision; project administration. **Sophia Tsabouri:** Investigation; writing – review and editing. **Antoine Deschildre:** Investigation; writing – review and editing. **Ismail Ogulur:** Investigation; writing – review and editing. **Susanne Lau:** Investigation; writing – review and editing. **Antonio Nieto Garcia:** Investigation; writing – review and editing. **Maria Jose Torres:** Investigation; writing – review and editing. **Liam O'Mahony:** Investigation; writing – review and editing. **Philippe Eigenmann:** Conceptualization; investigation; funding acquisition; writing – review and editing; data curation; supervision; resources; project administration. **Mohamed Shamji:** Investigation; writing – review and editing. **Mark Kačar:** Investigation; writing – review and editing. **Milena Sokolowska:** Investigation; writing – review and editing.

## FUNDING INFORMATION

Clemens von Pirquet Foundation.

## CONFLICT OF INTEREST STATEMENT

R.C., M.V., I.T., R.F.S., M.J.F., M.K., A.K., J.L., U.R., M.A., F.C., J.H., A.N.G., I.O., L.O., M.S., M.J.T., S.T., P.E. have no conflict of interest to declare. M.G. reports personal fees from Sanofi and Thermo Fisher Scientific. C.A.A. has received research grants from the Swiss National Science Foundation, European Union (EU CURE, EU Syn‐Air‐G), AO Research Institute, Davos, Novartis Research Institutes, (Basel, Switzerland), Stanford University (Redwood City, USA), Harvard University, TH Chan Institute of Epidemiology (Boston, MA, USA), Seed Health (Boston, USA) and SciBase (Stockholm, Sweden); is the Co‐Chair for EAACI Guidelines on Environmental Science in Allergic diseases and Asthma; Chair of the EAACI Epithelial Cell Biology Working Group is on the Advisory Boards of Sanofi/Regeneron (Bern, Switzerland, New York, USA), Stanford University Sean Parker Asthma Allergy Center (CA, USA), Novartis (Basel, Switzerland), Glaxo Smith Kline (Zurich, Switzerland), Bristol‐Myers Squibb (New York, USA), Seed Health (Boston, USA) and SciBase (Stockholm, Sweden); and is the Editor‐in‐Chief of Allergy. M.B. received honoraria from Pharming Group N.V. and Takeda. M.S. has received research grants from the Swiss National Science Foundation (SNSF nr 310030_189334/1; 320030E_224154; 320,030–236,264), GSK, Novartis, Stiftung vorm, Bündner Heilstätte Arosa, Thermofisher, OM Pharma, and TMG, speaker's fee from AstraZeneca, and consulting fee from Roche. A.D. received speaker/consulting fees from Aimmune Therapeutics, ALK, AstraZeneca, Celltrion, DBV Technologies, GSK, Novartis, Regeneron Pharmaceuticals Inc., Sanofi, Stallergenes Greer, Viatris, outside the submitted work. He is a member of the steering committee of the European Respiratory Society Clinical research collaboration SPACE (Severe Pediatric Asthma Collaborative Europe) that has been or is funded by Novartis, AstraZeneca, Sanofi, and Celltrion. S.L. reports to receive honoraria for lectures and/or advisory boards from Sanofi, ALK, Allergopharma, DBV technologies, Lilly, AstraZeneca, Leo Pharma, Almirall, Bencard, Viatris, Galderma, GSK. M.I.D.‐D. acknowledges the support of the European Respiratory Society Fellowship ‘Long‐Term Research Fellowship (LTRF) 2025’.

## Data Availability

Data sharing not applicable to this article as no datasets were generated or analysed during the current study.
